# Chlorine

**Published:** 1856-01

**Authors:** G. Watt

**Affiliations:** Xenia, Ohio.


### ARTICLE XII.

*Chlorine.* (CL=35. 42.) By G. WATT, D. D. S., M. D., Xenia, Ohio.

CHLORINE, under ordinary circumstances, is a greenish yellow gas. Its name is derived from the Greek word which expresses its color. When reduced, by pressure, to one-fifth its ordinary volume, it becomes a liquid, whose density is 1.33. It has not yet been congealed. The density of the gas is 2.44, hence it is 2½ times as heavy as air.

Chlorine was discovered in 1774, by Scheele, who called it "*dephlogisticated marine acid*," and was supposed to consist of muriatic acid and oxygen. About 1809 or 1810, it was classed by Davy, with the simple bodies, and this position is now universally assigned it by chemists. Chlorine is usually obtained by the action of hydro-chloric acid on binoxyd of manganese.

Equal parts of the two ingredients may be put into a retort and heat applied. The gas may be collected from the discharging tube, over warm water; but it is better to collect it, in dry bottles, by displacement of air. The reaction which takes place is as follows:

| | | | |
|---|---|---|---|
| 2 eq. Hydchl. acid, | 1 eq. Cl. - - - | | Is set free, |
| | 1 eq. Cl. - - - | | 1 eq. M. Cl. |
| | 2 eq. H. | 2 eq. water. | |
| 1 eq. Binox. Manganese | 2 eq. ex. | | |
| | 1 Mangan - | 1 eq. | |

The affinities which determine these changes, are the mutual attractions of oxygen and hydrogen, and of chlorine and manganese.

Distilled or recently boiled water dissolves twice its volume of chlorine, and yields it again when heated. This solubility renders it impracticable to preserve it over water, and its affinity for mercury prevents its being collected over that metal.

Chlorine has an astringent taste and a disagreeable odor. It is one of the most suffocating gases, causing spasm of the glottis and great irritation, even when considerably diluted. Light produces no effect upon it when dry, but if water be present, its hydrogen combines with the chlorine, forming hydrochloric acid. Hence the necessity of keeping solutions of chlorine in the dark is manifest.

Chlorine has powerful affinities. It unites energetically with hydrogen, and on this property depend many of the chemical phenomena to which it gives rise. An explosion always takes place when a lighted taper is plunged into a mixture of these two gases. It acts powerfully on the metals, readily converting them entirely into chlorides. Most of them combine with it even in the cold, and so energetic is the combination with many, that the temperature rises to ignition. Many substances take fire when thrown in a finely powdered state into a bottle filled with chlorine, and hence it may be called a supporter of combustion.

Chlorine is, indirectly, a most powerful oxydizing agent. Thus, when chlorine and a body which has a strong affinity for oxygen are brought in contact, water is usually resolved into its elements, its hydrogen uniting with the chlorine, and its oxygen with the other body.

When a compound of chlorine and an inflammable substance is exposed to a galvanic current, the inflammable body goes to the ——, and the chlorine to the $+$ pole. A close analogy is thus established between oxygen and chlorine, both are negative electrics, and both supporters of combustion.

Chlorine is extensively used in the arts for bleaching linen and cotton fabrics, and, in general, for destroying animal and vegetable colors. These coloring matters, like all other organic substances, are composed of carbon, hydrogen, oxygen, and sometimes of nitrogen. Chlorine generally decomposes them by appropriating their hydrogen, to form hydrochloric acid. In some cases, however, it seems that there is no bleaching, in the absence of moisture. It is probable then, that the oxygen, liberated by the decomposition of the water, is the bleaching agent.

Chlorine is extensively used for fumigation. For destroying miasmatic, noxious effluvia, and putrid odors, it is the best agent known. It acts as a poison on the animal system, both when inhaled and when swallowed. It should, therefore, be handled with due caution.

The compounds of chlorine, which are not acid, are called chlorides. Some of its combinations are interesting and important to the dentist. A few only will be noticed at present, and these without much regard to system.

*Hydrochloric Acid*, ($HCl.=36.42.$)

A concentration of this acid, is the *muriatic*, *chlorohydric*, or *hydrochloric* acid of commerce. In its purer form of gas, it was discovered by Priestly, in 1772. It is conveniently obtained by putting a portion of the hydrochloric acid of commerce in a glass flask, and heating it till the liquid boils, when the gas is freely evolved, and may be collected over mercury. It cannot be collected over water. It is also prepared by the action of concentrated sulphuric acid on common salt. The gas is freely disengaged by the application of heat. In the former case, the hydrochloric acid, previously dissolved in the water, is simply expelled from it by heat. The reaction in the latter case is represented by the following equation: $Na.\ Cl.+SO3,\ HO=Na.O+HCL$; or it may, perhaps, be better understood by the following diagram:

Chloride of sodium { Chlorine, / Sodium } — Chlorine and Sodium … } Hydrochlor acid.

| | | | | |
|---|---|---|---|---|
| Chloride of sodium | Chlorine, | | | Hydrochlor acid. |
| | Sodium | Soda | | |
| Sulph. acid of commerce. | water | oxygen | | |
| | | Hydrogen | | |
| | Sulph. acid . . . . . . . . . | | | Sulph. of Soda. |

Hydrochloric acid may be generated by the direct combination of its elements; a mixture of equal volumes of the two gases unite by explosion on the introduction of flame, a red hot body, spongy platinum, or by the passage of an electric spark. Even in diffused light they combine, but without explosion.

Hydrochloric acid is colorless, gives off copious fumes, which are formed by the acid uniting with the water of the atmosphere. It has a pungent odor and an acid taste, is irrespirable, exciting violent spasm of the glottis, extinguishes burning bodies, and is not inflammable.

It is not chemically changed by heat, but is readily decomposed by galvanism, hydrogen appearing at the —, and chlorine at the + pole. A striking property of the gas is its attraction for water, which, at 32° absorbs more than 500 times its volume of it. Ice is instantly liquified by it. The density of the liquid acid concentrated in the cold, is 1.21.

The hydrochloric acid of commerce is seldom pure, generally showing a yellowish tinge, caused by the presence of iron, and it frequently contains a small quantity of sulphurous acid. It is readily purified by distillation. The sulphurous acid may be converted into the sulphuric, by passing a few bubbles of chlorine through the solutions, and the sulphuric is precipitated, as sulphate of baryta, by chloride of barium.

A solution of the pure acid is perfectly colorless. In cases of poisoning by hydrochloric acid, the antidotes are chalk, whiting, magnesia, soap, &c.

*Compounds of Chlorine and Oxygen.*—Both chlorine and oxygen manifest an energetic attraction for most elementary substances, yet they have but a slight affinity for each other, they are consequently not found in nature, in a state of combination. They cannot be made to combine directly, and when united, very slight causes separate them.

They form several combinations, five of which are well ascertained, and are possessed of acid properties. These are:—

1. Hydrochlorous acid, $ClO$.
2. Chlorous acid, $ClO3$.

3. Hypochloric acid, $ClO_4$.

4. Chloric acid, $ClO_5$.

5. Perchloric acid, $ClO_7$.

Of these, we will notice at present, only.

*Chloric Acid*, ($ClO_5 = 75.42$.)

When a strong solution of potassa is saturated with chlorine, white crystals of chlorate of potassa are separated after standing, while a large quantity of chloride of potassium, and a small quantity of chlorate of potassa, remain in solution. The reaction which takes place is as follows:

$$Cl. + 6KO = 5\ KCl. + KO,\ ClO_5.$$

To purify the chlorate, dissolve it in boiling water—the greater part of it will be deposited in crystals, as the liquid cools.

All the compounds of chlorine and oxygen are prepared from the chlorate of potassa. Chloric acid is prepared by adding to a solution of chlorate of potassa, hydro-fluosilicic acid. The gelatinous silico-fluoride of potassium is precipitated, and chloric acid remains in solution. But as the silico-fluoride is a transparent precipitate, it is difficult to know when the potassium is entirely precipitated, and the hydro-fluosilicic acid, is therefore likely to be used in excess, hence the liquid, containing hydrofluosilicic, and chloric acids should be filtered, and saturated with a solution of baryta, which forms an insoluble hydro-fluosilicate with the one, and a soluble chlorate with the other. The liquid is again filtered and evaporated, and the chlorate of baryta is obtained in crystals. The chloric acid may then be separated by dissolving the chlorate in water, and adding sulphuric acid, as long as a precipitate is formed. The sulphate of baryta may be separated on a filter, and the liquid which contains only chloric acid, may be evaporated to the proper consistence, under the receiver of an air pump.

Chloric acid is already decomposed by heat, or by the presence of de-oxydizing agents. At a little above 100°, it is converted into perchloric acid, ($ClO_7$,) and chlorous acid, ($ClO_3$.) At a still higher temperature it is resolved into its elements. Sulphuric acid deprives it of oxygen, with the formation of sulphuric acid and the evolution of chlorine.

By mixing chloric with hydrochloric acid, chlorine is copiously liberated, as may be illustrated by the following equation:

$$5HCl + ClO5 = 5HO + 6Cl.$$

This mixture is an admirable solvent for gold, platinum, &c., but the inconvenience of preparing, and the difficulty of preserving the chloric acid, renders its use impracticable, in most cases.

*Aqua Regia.*—Chlorine is also freely disengaged from a mixture of nitric and hydrochloric acids. This well known mixture was called by the alchemists, aqua regia, (royal water,) from its power to dissolve gold, which they regarded as the *king* of metals. It may be made by mixing one volume of nitric, with 2, 3, or 4 volumes of hydrochloric acid.

When aqua regia acts on any metal, the following reaction takes place between the two acids of which it is composed:

$$NO\,5 + 2\,HCl = NO3 + 2\,H\,O + 2\,Cl.$$

A metal placed in this liquid, therefore, meets chlorine in its nacent state, and is, in consequence, rapidly dissolved in the state of chloride.

The metallic chlorides might be considered with more propriety, perhaps, under the various metals, but as this is an isolated article, we will notice a few of them here.

*Chloride of Sodium.* (Na. Cl. = 58.7.)

This well known table salt, may be formed directly, by heating sodium in hydrochloric acid gas, by burning the metal in chlorine, or by neutralizing soda with hydrochloric acid. It is the chief saline ingredient of sea-water, and exists as a mineral under the name of rock-salt, and it is contained in many saline springs. Obtained from any of these sources it contains traces of sulphate of magnesia and lime and chloride of magnesium. These may be precipitated as carbonates by boiling a solution of the salt with an excess of carbonate of soda, then by filtering the liquid, and neutralizing with hydrochloric acid, pure chloride of sodium is obtained.

When a solution of chloride of sodium evaporates spontaneously, the crystals are regular cubes, but when the process is rapidly conducted they are hollow four-sided pyramids, formed by the adhesion of minute cubes. They contain no water of crystalization.

This salt is also found in the organized kingdom, as in saline plants, and in the blood, saliva, urine, etc., of man, and many of the lower animals.

Its general use is well known. The dentist uses it in the laboratory to precipitate silver, as a chloride, and some use it as a dentifrice—a practice of at least doubtful utility.

*Chloride of Zinc.* (Zn. Cl.=67.72.)

When minutely divided zinc is introduced into chlorine gas, they combine with the evolution of heat and light, and this salt is formed. It is readily and cheaply prepared by dissolving the metal, or its oxyd, in hydrochloric acid, evaporating to dryness, and fusing the residue in a narrow necked glass vessel.

Chloride of zinc is a grayish-white, soft, semi-transparent mass. It is soluble in water, alcohol and ether. It fuses a little above 212°, and sublimes at a red heat. It is highly deliquescent.

Its local action on living tissue is that of an escharotic. This action depends principally on its affinity for albumen and gelatine. As a caustic it is exceedingly active, decomposing the organic tissues as rapidly as nitrate of silver, extending its action at the same time, to parts more deeply situated.

This salt is extensively used as an escharotic application to inflamed dentine. Of course it should never be used when it is practicable to restore the inflamed part to its normal condition. The subjacent tissues are left in a healthier condition after its use than after nitrate of silver, and some other caustics, but the loss of substance is greater.

The oxy-chloride of zinc, made by simply dissolving zinc in hydrochloric acid, is a good flux for almost any of the soft solders which the mechanical dentist may wish to use incidentally, in the laboratory.

*Chloride of Silver.* (Ag. Cl.=143.42.)

This compound, known as horn-silver, by mineralogists, may be generated by heating silver in chlorine gas, or by mixing hydrochloric acid, or any soluble chloride, with a solution of nitrate of silver. The most convenient precipitant is the chloride of sodium, or common salt. When common gold coin, or any alloy containing silver, is dissolved in aqua regia, the silver is found at the bottom of the vessel, in the form of chloride. When first precipitated it is white, but by exposure to light, it soon becomes almost black.

It is insoluble in water, and but sparingly soluble in the stronger acids. It is readily dissolved in ammonia. It fuses at about 500°, and is then a yellow liquid, which on solidifying becomes a semi-transparent horny mass, identical with the native horn-silver. It bears almost any degree of heat without change, but is readily decomposed by hydrogen. A convenient method of effecting this is to place the chloride in a glass jar, add granulated zinc and dilute sulphuric acid. The hydrogen thus disengaged seizes the chlorine, forming hydrochloric acid, which escapes as gas, leaving the silver in minute granules, in the bottom of the jar. The zinc must all be dissolved, by the sulphuric acid, and the sulphate of zinc being soluble, is easily removed by washing and decantation. This decomposition is thus illustrated.

$$Ag.\ Cl. + H. = Ag. + HCl.$$

*Chlorides of Gold.*—When gold is dissolved in aqua regia and the solution sufficiently concentrated by evaporation, very fusible, ruby red prismatic crystals are obtained. By heating these crystals to about 600°, we obtain an insoluble, yellow substance, the

*Protochloride of Gold.* (Au. Cl.=234.62.)

By boiling in water, the protochloride is resolved into the terchloride, and metallic gold. At a red heat it loses all its chlorine, and metallic gold remains. It is unimportant to the dentist.

*Terchloride of Gold.* (Au. Cl. 3 = 305.46.)

The crystals above described are composed of the terchloride of gold. In this form, gold is usually, and most conveniently dissolved. It is readily decomposed by protosulphate of iron, by which the metal is thrown down, as a brown powder, and the solution contains sesquisulphate of peroxyd, and terchloride of iron. The reaction is as follows:

6 (Fe.OSO 3)+Au. Cl.3=2(Fe. 2 O 3 3SO 3.)+Fe.2 Cl3+Au.

Slight traces of iron sometimes adhere to the precipitate but they are easily removed by washing with dilute hydrochloric acid.

Nitrate of mercury, most of the metals, sulphurous, phosphorous, and oxalic acid, also decompose it, and precipitate the gold.

The terchloride of gold is soluble in water, alcohol, and ether. The ethereal solution is an admirable escharotic application to inflamed dentine. The indications for its use are the same as those for chloride of zinc. It is conveniently prepared by evaporating the ordinary solution of gold, till the formation of crystals commences; then dissolve the residue in water, and add, in a vial, an equal volume of sulphuric ether and agitate. Two fluids will result, the lighter of which is the ethereal solution of terchloride of gold. It may be poured into a separate vial, closely corked, and set in a dark place for future use. The gold may be precipitated from the remaining solution with sulphate of iron.

When the ethereal solution is applied to a tooth, the terchloride is almost instantly decomposed, and the three equivalents of chlorine thus liberated, act with the energy peculiar to the nascent state, and the result is that the vitality of the inflamed dentine is instantly destroyed, and with far less pain than results from chloride of zinc, or any other escharotic that I have used. It is probable also that the presence of the ether has some influence over the pain.—*Dental Register.*

